# Death of Sylvester Graham

**Published:** 1851-11

**Authors:** 


*Death of Sylvester Graham.* — Most of our readers in this part of the country are familiar with the name of this individual, who some years since made himself notorious in urging upon the community the system of light and exclusively vegetable diet to which his name was given. He has lately died at Northampton, in this state, at the age of about fifty. The Gazette, of that place, states that his health had been gradually failing for the last year, and he had suffered much from rheumatism in his hands and feet. "A post-mortem examination disclosed no disease in the system, which, in the opinion of the medical examiners, was sufficient to produce his death; and the immediate cause of his decease is thought to be the use, contrary to the advice of his physician and friends, in the extreme exhaustion of the system, of Congress water and a tepid bath." — *Ibid.*

